# Clinical Outcomes of CAD/CAM (Lithium disilicate and Zirconia) Based and Conventional Full Crowns and Fixed Partial Dentures: A Systematic Review and Meta-Analysis

**DOI:** 10.7759/cureus.37888

**Published:** 2023-04-20

**Authors:** Gunjan S Aswal, Renu Rawat, Dhara Dwivedi, Nitin Prabhakar, Vinod Kumar

**Affiliations:** 1 Dentistry, The University of the West Indies, St. Augustine, TTO; 2 Dentistry, Hawassa University, Hawassa, ETH; 3 Dentistry, Jimma University, Jimma, ETH

**Keywords:** survival analysis, clinical success, clinical performance, fixed partial dentures, all ceramic, full crowns, cad/cam

## Abstract

Although CAD/CAM (computer-aided design/computer-aided manufacturing) restorations act as a favorable alternative to conventional metal-ceramic restorations for fixed dental prostheses, little is known about their intermediate and persistent clinical performance. This systematic review and meta-analysis aimed to assess the clinical performance in terms of biological, technical, and esthetic aspects and the survival and success ratios for single full crowns (SFCs) and fixed partial dentures (FPDs) fabricated by CAD/CAM and conventional techniques and according to the materials used (zirconia {ZC} and lithium disilicate {LD}). The population, intervention, control, outcome, and study design (PICOS) strategy was used to electronically search key terms in the PubMed, Cochrane Library, Embase, and Wiley Online databases for randomized control trials (RCTs) and cohort studies. The bias risks for RCTs and cohort studies were assessed using the Cochrane collaboration tool and the Newcastle-Ottawa Scale (NOS). Meta-analysis was performed using Rev5 from Cochrane. A total of 13 studies reporting on 1598 restorations in 1161 patients with a mean observation period of 3.6 years (minimum-maximum: 1-9.3 years) met the inclusion criteria. Meta-analysis of the included studies indicated that CAD/CAM manufacturing resulted in 1.17, 1.14, and 16.88 (95% CI: 0.64-2.17, 0.86-1.52, 7.59-37.56) higher biological, technical, and esthetic complications than conventional manufacturing of restorations. However, the difference was significant for esthetic complications only (p<0.00001). A significant difference was observed for all biological, technical, and aesthetic aspects between SFCs and FPDs (odds ratio {OR} = 2.61 vs. 1.78, 95% CI: 1.92-3.56 vs. 1.33-2.38; p<0.00001). The survival ratio of SFCs was 2.69 (95% CI: 1.98-3.65), significantly higher compared to the 1.76 (95% CI: 1.31-2.36) of FPDs (p<0.00001). The success ratio of FPDs at 1.18 (95% CI: 0.83-1.69) was significantly lower compared to SFCs at 2.36 (95% CI: 1.68-3.33). The clinical performance of LD with 2.42 (CI: 1.16-5.03) was significantly higher compared to ZC with 2.22 (CI: 1.78-2.77) (p<0.00001). The biological, technical, and aesthetic behaviors showed similar clinical outcomes between the CAD/CAM and conventional groups. LD could be a good alternative to zirconia, but its intermediate or persistent clinical performance needs to be evaluated. Overall, zirconia and CAD/CAM techniques must evolve further to outclass the conventional techniques used in the fabrication of SFCs and FPDs.

## Introduction and background

The transitioning dental care requirements, the evolution of technology and progress in dental materials in the last few years have promoted the increasing use of rapid and convenient digital clinical workflows based on computer-aided design (CAD) or computer-aided manufacturing (CAM) technology over the conventional materials & technology, with an established record of clinical success and compatibility with human tissues, to support high-performance restoration arrangements in modern dental clinics [1,2]. These CAD/CAM workflows have revolutionized treatment approaches by enabling the designing and development of dental restorations, including single full crowns (SFCs) and fixed partial dentures (FPDs) for chairside applications [3]

Some of the most apparent benefits of the CAD/CAM technique over conventional manufacturing of restorations include the streamlined production work in the laboratory, single consultation and the requirement of less treatment time [4,5]. CAD/CAM has a key benefit in that this technique stores clinical information by electronic means and allows the remake of a damaged restoration without a consultation [6]. Besides, the higher precision and workflow digitalization using CAD/CAM is considered reliable when it comes to achieving esthetic expectations of patients in a less operator-dependent manner [5]. The clinical workflows based on conventional manufacturing techniques use metals alone or in combination with ceramics for fixed dental prostheses (FDPs), including SFCs and FPDs, which generally lack esthetic benefits and require a more invasive tooth arrangement and a prolonged manufacturing time. Thus, only-ceramic options have progressively become a promising alternative approach over metal-based materials for SFCs and FPDs [7]. Considering the demands of customers as well as dentists in the restorative dentistry field, the CAD/CAM all-ceramic materials have been receiving increasing attention as an alternative to conventional materials owing to their esthetic, technical and biological features [8,9]. In this context, CAD/CAM systems are considered to be viable options owing to their durability, compatibility with human tissues, esthetic characteristics and survival rates [3].

Technology-enabled digital workflows facilitate the development of ceramics like lithium disilicate (LD) and zirconia (ZC), among others [10]. LD is one of the most extensively used glass-ceramic materials because of its successful clinical performance and high acceptance among patients and clinicians for chairside applications. ZC is one of the highly promising oxide ceramics for full crowns owing to its high resistance to cohesive fractures, high mechanical reliability and proper architectural framework [11]. However, it has a less attractive aesthetic feature. From a clinical perspective, in the last 10 years, ZC has gained more attention in the context of metal-free, ceramic material predominantly used for restoring natural teeth as well as implants with full crowns [12]. Thus, the outcome of these materials must be examined to establish the workflow appropriate for each unique dental case since a correct selection of the technique-based fabrication material is a key factor influencing the success or failure of dental restorations [1].

Despite the significant increase in the application of CAD/CAM materials over conventional materials for full crowns and FPDs, there lies a key concern in the clinical performance of the CAD/CAM-all ceramic materials [13]. CAD/CAM all-ceramic materials are not without complications in terms of internal fit, adhesion issues, marginal integrity loss, microleakage, and possible tooth hypersensitivity compared to conventionally manufactured materials [14]. Besides, CAD/CAM ceramic materials might result in improper veneer discharge, thickness, surface damage and cooling rate errors due to veneer and framework differences [15]. Also, the differences in manufacturing techniques of all ceramics present a concern as to which ceramic material presents a higher clinical performance and survival rate [16]. The estimated survival rate of LD-based crowns fabricated using a conventional technique for five years ranges from 90.7% to 96.6%, while the outcome of CAD/CAM manufactured LD-based FPDs is reported to be nearly 91.6% [7]. The use of ceramic materials for FPDs and crowns offers limited evidence in terms of clinical performance, survival, success and failure rates in each dental case [9,17]. Moreover, information regarding the clinical performance of CAD/CAM-based restoration materials compared to conventionally manufactured materials has been scarce and scattered. To date, evidence regarding the clinical performance of LD and ZC is still limited and often debatable, highlighting the need for further studies to precisely outline the clinical indications and durable performance of such CAD/CAM-based ceramics [18,19]. Furthermore, information regarding which fabrication technique is the best, the preference being primarily guided by clinical outcomes, is still controversial [20]. Thus, the new range of ceramic CAD/CAM materials offered for FPDs and crowns indicates the necessity for methodical evidence that clinically assesses the outcome of these fabrication techniques and the materials used. Therefore, the present systematic review and meta-analysis aimed to assess the clinical (biological, technical and esthetic) performance, survival and successes of LD- and ZC-based CAD/CAM and conventional SFCs and FPDs reported in randomized controlled trials (RCTs) and cohort studies. Thus, the present study was conducted to address the following population, intervention, control, outcome, and study design (PICOS) questions: (a) Whether the clinical performance of CAD/CAM restorations is comparable with that of conventionally manufactured (using lost wax technique) SFCs and FPDs? (b) Whether the survival and success ratios of all biological, technical and esthetic aspects of SFDs and FPDs made out of LD- and ZC-based CAD/CAM restorations comparable with that of conventionally manufactured ones? (c) Which CAD/CAM ceramic material between LD and ZC exhibited a better clinical performance?

## Review

Methods

The PRISMA (preferred reporting items for systematic reviews and meta-analyses) procedures and checklists were followed in the present study [21]. The components of PICOS framework are as follows: P - Patients with SFCs and FPDs; I - Patients with at least one LD- or ZC-based SFC or FPD fabricated using CAD/CAM; C - Patients with at least one SFC or FPD fabricated using conventional material; O - Clinical performance, survival, and success ratios of all biological, technical, and aesthetic aspects; S - RCT or cohort studies (prospective and retrospective).

Search Strategy

**Figure 1:**
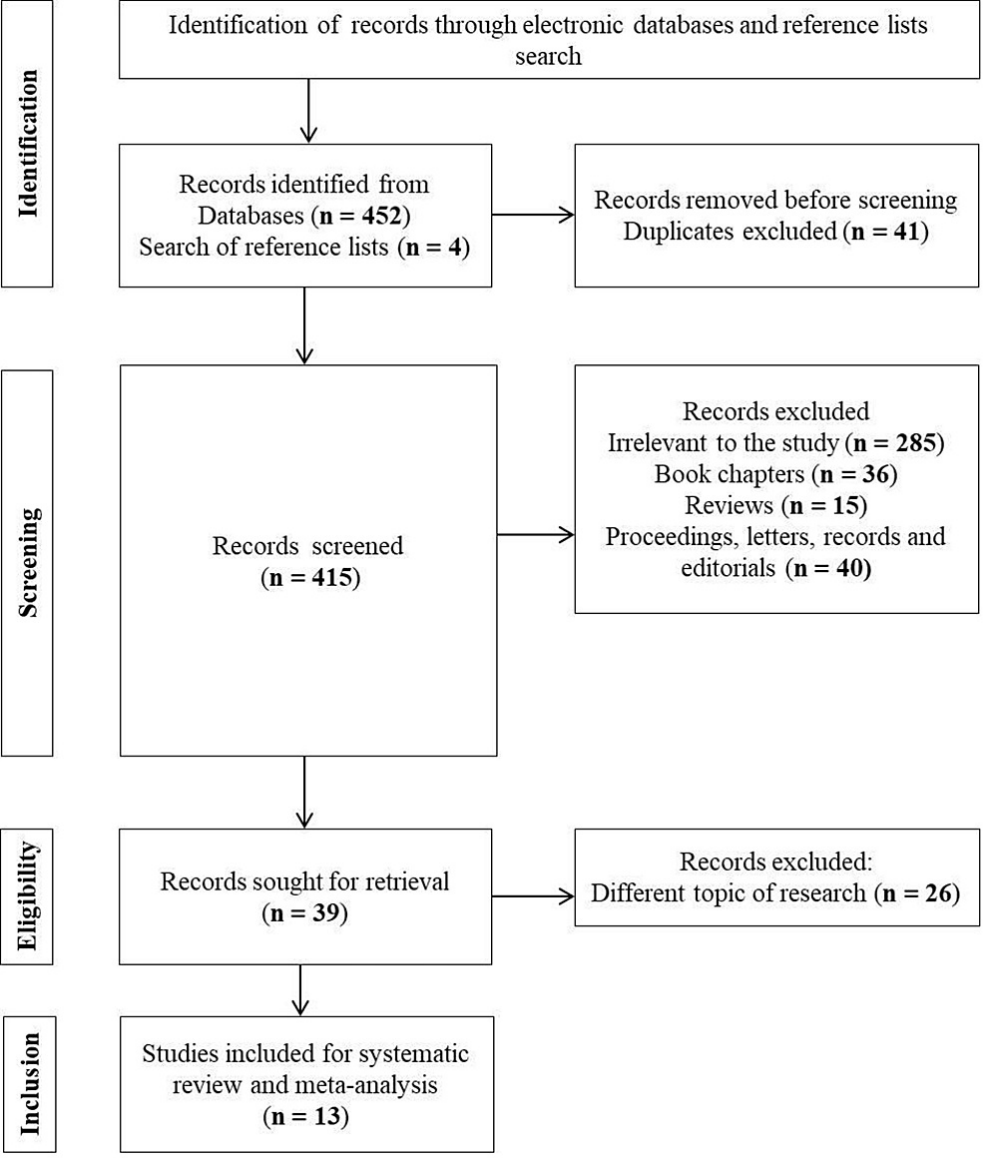
PRISMA chart representing the selection of articles with inclusion and exclusion criteria PRISMA: Preferred reporting items for systematic reviews and meta-analyses

The present study followed a systematic approach, where the first step included searching for keywords. A methodical keyword search strategy was conducted in major electronic databases like PubMed, Cochrane, Embase, and Wiley Online, focusing on articles related to the clinical performance of LD- or ZC-based CAD/CAM and conventionally manufactured SFCs or FPDs. Articles with the following Medical Subject Heading (MeSH) terms were searched: crowns, dental crowns, full crowns, single crowns, fixed partial dentures, FPDs, fixed dental prostheses, FDPs, computer-aided design, computer-aided manufacturing, CAD, CAM, computers, dentistry, dental milling, CEREC, ceramics, dental ceramics, lithium disilicate, zirconia, LD, ZC, conventional manufacturing, laboratory manufacturing, heat-pressed, metal ceramic, clinical performance, success, survival rate, follow-up study, comparative study, clinical trial, randomized controlled trials, cohort study. The boolean words "OR" and "AND" were used. The following search was carried out:

(‘Crowns’ OR ‘dental crowns’ OR ‘single crowns’ OR ‘full crown’ OR ‘fixed partial dentures" OR "FPDs" OR ‘fixed dental prostheses" OR ‘FDPs’) AND (‘computer-aided design’ OR ‘computer-aided manufacturing’ OR ‘CAD’ OR ‘CAM’ OR ‘computer’ OR ‘dentistry’ OR ‘dental milling’ OR ‘CEREC’ OR ‘ceramics’ OR ‘dental ceramics’ OR ‘lithium disilicate’ OR ‘zirconia’ OR ‘LD’ OR ‘ZC’) AND (‘conventional manufacturing’ OR ‘ laboratory manufacturing’ OR ‘heat-pressed’ OR ‘metal ceramic’) AND (‘clinical performance’ OR ‘success’ OR ‘survival rate’) AND (‘follow-up study’ OR ‘comparative study’ OR ‘clinical trial’ OR ‘randomized controlled trials’ OR ‘cohort study’). The electronic database search yielded 452 articles and four were identified through a manual search of reference lists (Figure 1).

Eligibility (Inclusion and Exclusion) Bases

The stepwise selection of the 452 articles obtained was based on the relevance of titles, abstracts, assessment of the main body, and close examination against the following preset inclusion criteria: randomized controlled trials (RCTs) and cohort studies (retrospective and prospective) on humans published in dental journals were considered. Studies assessing the clinical performance of either LD- or ZC-based CAD/CAM, and conventionally manufactured SFCs or FPDs on patients above 18 years of age were included. Further, the search aimed for articles available in the English language. Book chapters, systematic and literature reviews, proceedings, records, in vitro studies, and editorials were excluded. Studies that considered CAD/CAM manufacturing of restorations other than SFCs or FPDs were filtered out. Studies that considered CAD/CAM manufacturing with materials other than LD or ZC were excluded.

Data Extraction

From each study, data on the following variables were obtained: author(s), study design, the total number of patients, mean patient age, number, and type of restorations, follow-up duration, manufacturing technique, failure, survival, success, clinical performance, and conclusive findings.

Assessment of Outcomes

The clinical performance of the restorations in the selected studies was assessed using the modified version of the United States Public Health Service (USPHS) standard [22]. This standard is based on the evaluation of the clinical performance in terms of esthetic attributes (surface roughness, luster, marginal staining, color match, and clearness), technical characteristics (marginal adaptation, anatomic form, material fracture, chipping, and retention loss), and biological characteristics (secondary caries, endodontic response, post-surgery sensitivity, recurrence, oral vitality, and periodontal parameters). The restoration outcomes were evaluated based on the four categories from A-D, where A (Alpha) indicates restoration in excellent condition concerning the factor under consideration and a long-term survival expectancy; B (Bravo) indicates restoration in balanced condition with the possibility of a replacement requirement in the future; C (Charlie) indicate restoration failure or surrounding tissue failure; and D (Delta) indicates restoration failure concerning the factor under consideration. "Clinical performance" was indicated by biological, technical, and esthetic outcomes [23]. "Success" was indicated by successful restorations that meet high-quality standards without any complications and "survival" was indicated by no requirement of restoration replacement although the restoration had deteriorated due to complications, and "failure" was indicated by any need for restoration replacement [24].

Bias Assessment

The methodological quality assessments of the studies were done using the Cochrane collaboration tool for RCTs, evaluating bias risks in seven domains such as the random assignment of respondents into groups, concealment of the randomization sequence, blinding of participants and personnel, blinding of outcome assessment, selective reporting, incomplete outcome data, and other sources of bias [25]. The overall risk of bias for each domain reported by the selected studies was assessed as ‘yes’, ‘no’, or ‘unclear’ for a low, high, or unclear possible risk of bias. Besides, the methodological quality of the retrospective and prospective studies was assessed using the Newcastle-Ottawa Scale (NOS) in three domains, such as sample selection, comparability, and outcome of the study [26]. The NOS consists of eight items, resulting in a total score of 9. Studies yielding a score between 3 and 4 (selection), 1 and 2 (comparability), and 2 and 3 (outcome) are considered high-quality studies with a low bias risk.

Data Analysis

To conduct the meta-analysis, all materials across the articles were pooled due to sampling considerations or missing information in a random and fixed-effects model. Possible publication bias across the studies was detected using Egger’s test, and variation due to statistical heterogeneity among the studies was detected using the I^2^ statistic. I^2^ score ˃ 70% was highly heterogenic [27]. Results were graphically represented using forest and funnel plots. The restorations were considered the statistical unit. Here, statistical significance was considered at p < 0.05. All analyses were performed using Rev5 from Cochrane.

Results

Identification of Studies and Descriptions

Key term searches in the electronic databases (PubMed, Cochrane, Embase, and Wiley Online) and manual searches of reference lists identified 456 articles. Of the 415 articles screened, 39 were selected for assessment of the main body. A final total of 13 studies were included in the systematic review and meta-analysis based on the inclusion criteria. The elucidation of each study is outlined in Table 1.

**Table 1:** Summary of included studies CAD: Computer-aided Design; CAM:  Computer-aided Manufacturing; MC:  Metal Ceramic; HP:  Heat Pressed; FDP:  Fixed Dental Prostheses; FPD:  Fixed Partial Denture; SFC:  Single Full Crown; PFM:  Porcelain-fused-to-Metal; LD:  Lithium Disilicate; ZC:  Zirconia; RCT:  Randomized controlled Trial

Author (s)	Study design	Patients	Mean age (years)	Restoration no	Restoration type	Follow-up (years)	Manufacturing technique	Clinical performance	Conclusive findings
Berrendero et al. [2]	Prospective	10	52.8	17	ZC-based posterior SFCs	4	CAD/CAM and putty-wash method	Technical (marginal fit) and biological (inflammation at occlusal and interproximal contact points) outcomes were better for CAD/CAM-based ZC SFCs compared to the control group	CAD/CAM-based restorations exhibited better clinical performance
Suarez et al. [9]	RCT	40	47	40	ZC and MC FPDs	5	CAD/CAM and conventional techniques	Minor technical complication (chipping) was observed for ZC-FPDs (20%). No biological complications were observed for both groups. ZC FPDs exhibited better anatomical form, marginal integrity and periodontal status compared to metal FPDs	A similar survival rate for the groups at the end of 5 years. However, the success rate of metal FPDs was better than ZC-FPDs.
Spehar and Jakovac [17]	RCT	44	41.5	22 (conventional) 22 (CAD/CAM)	Monolithic and layered LD based SFCs	1	CAD/CAM and conventional	The survival rate of LD SFCs fabricated using CAD/CAM was comparable to the conventional ones	Crowns manufactured using both CAD/CAM and conventional techniques had an optimum clinical fit
Christensen and Ploeger [28]	RCT	259	50	293	Zirconia (ZC) and metal framework fixed partial dentures (FPDs)	3	CAD/CAM and laboratory	Two of the ZC frameworks fractured at 36 and 40 months. No fractures were observed for metal frameworks	Five ZC CAD/CAM frameworks were significantly comparable to metal frameworks
Pelaez et al. [29]	RCT	37	-	40	ZC and metal-ceramic (MC) posterior- fixed dental prosthesis (FDPs)	4	CAD/CAM and conventional laboratory procedure	One biological (periodontal response) and one technical (chipping) complication in ZC-FDPs	ZC FDPs demonstrated a similar survival rate to MC FDPs for medium-term clinical use
Vigolo and Mutinelli [30]	Prospective	58	32	58	ZC and porcelain-fused to metal (PFM) posterior-FDPs	5	CAD/CAM and PFM	ZC-based FDPs exhibited more frequent technical problems in terms of extended fracture of the ceramic veneer	No significant difference in the clinical performance between ZC- and PFM-FDPs
Passia et al. [31]	RCT	223	42.7 (CAD/CAM) and 41.0 (conventional)	223	ZC and gold-based full crowns	5	123 (CAD/CAM) 100 (conventional)	Gold crowns exhibited better marginal integrity and less discoloration compared to the ZC-based crown	ZC-based crowns for posterior tooth dentures are not recommended
Brandt et al. [32]	Retrospective	319	50	425 MC and 88 ZC	ZC and MC FPDs	9.3	CAD/CAM and conventional	ZC FPDs exhibited higher success and survival rates than MC FPDs at the end of the observation period	No significant difference in the survival rate between ZC and MC FPDs
Sailer et al. [33]	RCT	58	52.7 (ZC) and 57.0 (MC)	76	ZC and MC FDPs	5	CAD/CAM and conventional	ZC FDPs exhibited inferior technical (chipping, marginal adaptation, and anatomical form), and biological (occlusal wear on restoration and periodontal status) outcomes compared to MC FDPs, being statistically non-significant	Similar clinical performance was observed for both the groups at the end of the 5 years
Cortellini and Canale. [34]	RCT	76	36	235	Monolithic LD full crowns	1.6	CAD/CAM and heat pressed (HP)	The esthetic outcomes were ideal	The use of CAD/CAM for restorations using full crowns can be considered a feasible option
Akin et al. [35]	RCT	15	29	15 (CAD/CAM) and 15 (HP)	LD based SFCs	2	CAD/CAM and HP	HP-ceramic crowns exhibited higher marginal discoloration and slight hypersensitivity than CAD/CAM crowns. But CAD/CAM exhibited lesser marginal adaptation	Overall satisfactory clinical performance
Muhlemann et al. [36]	RCT	10	51.2	5	Monolithic LD based SFCs	1	CAD/CAM and conventional	Conventionally manufactured crowns had better marginal adaptation	No significant difference in clinical performance were observed between both the groups
Morsy et al. [37]	RCT	12	31.5	24	Monolithic ZC FPDs	1	CAD/CAM and conventional polyether impression	ZC FPDs fabricated using CAD/CAM exhibited significantly better marginal and internal fits than the conventionally manufactured ones	FPDs fabricated using both techniques demonstrated an optimal clinical fit

Of all the 13 studies, nine were RCTs, three were prospective, and one was a retrospective study. The selected studies included SFCs (9 articles) and FPDs (4 articles). The number of restorations and the follow-up period ranged from 5 to 425 and 1 to 9.3 years, respectively. A total of 1598 restorations were placed in 1,161 patients with a mean age of 43.0 years in the included studies.The manufacturing techniques and materials analyzed were CAD/CAM (ZC and LD) and conventional porcelain-fused-to-metal (PFM). Seven of them compared and analyzed two different materials (ZC and metal) within the same study [9,28-33]. Four studies analyzed LD-based SFCs, differentiating their laboratory manufacturing processes (CAD/CAM and conventional) [17,34-36]. Only two studies evaluated ZC-based SFCs and FPDs, differentiating their laboratory manufacturing processes (CAD/CAM and conventional) [2,37]. Overall, ZC was used in nine articles, and LD was used in four articles for meta-analysis.

The description of the clinical outcomes for the selected studies is compiled in Table 2.

**Table 2:** Description of clinical outcomes of the included studies CAD: computer-aided design; CAM: computer-aided manufacturing; MC: metal-ceramic; HP: heat pressed; FDP: fixed dental prostheses; FPD: fixed partial denture; SFC: single full crown; PFM: porcelain-fused-to-metal; LD: lithium disilicate; ZC: zirconia;  NR: not recorded

Author	Clinical outcomes			
	Failure	Survival (%)	Success (%)	Clinical complications
Berrendero et al. [2]	Vertical root fractures and fractures at distal connectors after 2 years	76.5 (CAD/CAM and putty-wash)	60 (CAD/CAM) 40 (Putty-wash)	Biological: Occlusal contacts (Alpha): 11.5% (test), 6.5% (control) Technical: Interproximal contact points (Alpha): 13% (test) 7.5% (control) Marginal fit (Alpha): 16.5% (test), 11% (control) Primary retention (Alpha): 8% (test) 9% (control)
Suarez et al. [9]	No failures	100 (ZC and MC)	100 (MC) and 80 (ZC)	Biological: Periodontal inflammation: 5% (test), 15% (control) Technical: Chipping: 20% (test), 0 (control)
Spehar and Jakovac [17]	1 CAD/CAM crown fracture	100 (conventional) 95.5 (CAD/CAM)	100 (conventional) 99 (CAD/CAM)	Biological: Proximal contact defect: 0% (test), 9.1% (control) Technical: Ceramic fracture: 4.5% (test), 0 (control) Marginal gap: 4.5% (test), 0 (control) Esthetic: Marginal discoloration: 47.6% (test), 0 (control)
Christensen and Ploeger [28]	Two ZC FPDs fractured	100 (MC) 87 (ZC CAD/CAM)	100 (MC) 96 (ZC CAD/CAM)	Biological complication Dental caries: 1.3% (test), 1.1% (control) Technical complication: Framework fracture: 1.3% (test), 0 (control)
Pelaez et al. [29]	No fractures	100 (MC) and 95 (ZC)	Satisfactory restorations	Biological: Periodontal complications in one test sample Technical: Chipping was observed in two test samples
Vigolo and Mutinelli [30]	Replacement of two ZC-based FDPs	95 (PFM) and 85 (ZC)	NR	Technical: Extended fracture of veneering ceramic for test group (3), control (0)
Passia et al. [31]	Fracture of the ZC-based crowns	73.2 (ZC) and 92.3 (gold)	98 (gold-based) and 89.4 (ZC)	Biological: Higher frequency of secondary caries in test group compared to controls. Technical: Framework fracture: 25.1% (test), 1% (control) Marginal gap: in 5.6% (test), 1% (control) Esthetic: Marginal discoloration: 49.5% (test), 1% (control)
Brandt et al. [32]	A significantly higher risk of failure was observed in the ZC group	88.2 (ZC), 76.2 (MC)	73.1 (ZC) 66.7 (MC)	Biological: Apical osteitis: 1.1% (test), 5.2% (control) Root fracture: 1.1% (test), 0.5% (control) Perio-endo lesion: 0% (test), 0.2% (control) Technical: Chipping: 2.3% (test), 5.2% (control) Loss of retention: 15.9% (test), 1.9% (control) Insufficient restoration margin: 3.4%(test), 13.6% (control) Framework fracture: 4.5% (test), 1.4% (control) Esthetic failure: 0% (test), 0.5% (control)
Sailer et al. [33]	No failures	100 (ZC and MC)	75 (ZC), 89.7 (MC)	Biological: Occlusal wear: 25% (test), 10.35 (control) Periodontal pocket depth (PPD) (mean): 2.5 (test & control) Plaque control record (PCR): 13.8 (test), 12.9 (control) Bleeding on probing (BOP): 32.8 (test), 29.8 (control) Technical: Veneering chipping: 7.5% (test), 0 (control) Marginal gap: 17.5% (test), 10.3% (control) Anatomical shape (over or under contoured): 2.5% (test), 0 (control)
Cortellini and Canale. [34]	One crown from the control group fractured	99.5 (HP) 100 (CAD/CAM)	99 (HP) and 100 (CAD/CAM)	No biological or technical complications Control: One crown fractured
Akin et al. [35]	Minor fracture after 1 year but no restoration required.	100	100	Biological: Hypersensitivity: 1% (control), 0 (test) Technical: Marginal gap: 1% (control), 4% (test) Esthetic: Marginal discoloration: 3% (control), 0 (test)
Muhlemann et al. [36]	NR	100	62.5 (CAD/CAM) 70 (conventional)	Technical: Marginal gap: 70% (test) 10% (control) Loss of retention: 10% (test) 0% (control) Interproximal contact point missing: 20% (test) 0% (control)
Morsy et al. [37]	NR	NR	NR	Technical: Marginal gap: 30.9 (test), 40 % (control) Internal gap: 30.8 (test), 41.8 (control)

A total of seven studies reported that clinical complications in the CAD/CAM group exceeded those in the conventional group [17,28-31,33-36]. However, three studies reported that clinical complications in the conventional group exceeded those in the CAD/CAM group [32,35,37]. Only one study exhibited equal clinical complications for both groups [9]. The study by Berrendero et al. reported that the CAD/CAM group exhibited greater clinical performance compared to the conventional group [2]. Comparing CAD/CAM versus conventional SFCs, the most frequent biological complications with CAD/CAM SCs were periodontal inflammation, secondary caries, hypersensitivity, occlusal wear, and an approximate contact defect, and the most frequent technical complications were chipping, framework fracture, marginal gap, anatomical shape, loss of retention, and absence of an interproximal contact point. Marginal discoloration of the CAD/CAM based SFCs was the prevalent esthetic complication. Besides comparing CAD/CAM versus conventional FPDs, the frequent biological complications of conventional FPDs were periodontal and pulpal inflammations, dental caries, apical osteitis, and root fracture. The observed technical complications of CAD/CAM and conventional FPDs were framework fracture, chipping, loss of retention, insufficient restoration margin, and marginal and internal gaps.

Methodological Evaluation of Selected Studies

The methodological quality of the RCTs and the cohort studies is presented in Table 4 and Table 3 respectively.

**Table 3:** Newcastle–Ottawa scale for quality assessment of cohort studies 1) depiction of the exposed study; (2) selection of the non-exposed study; (3) identification of exposure; (4) demonstration that outcome of interest was not present at the start of the study; (5,6) comparability of cohorts based on the design or analysis; (7) outcome assessment; (8) was observation period sufficient for the occurrence of outcomes; (9) adequacy of the observation period

Study	Selection				Comparability	Outcome			Total
	1	2	3	4	5-6	7	8	9	
Berrendero et al. [2]	*	*	*	-	*	*	-	-	5/9
Vigolo and Mutinelli [30]	-	*	*	*	-	*	*	*	6/9
Brandt et al. [32]	*	*	*	*	*	*	-	*	7/9
Cortellini and Canale. [34]	*	*	*	-	*	*	-	*	6/9

**Table 4:** Cochrane tool for quality assessment of randomized controlled trials (RCTs) ^a^Randomization list; ^b^Split-mouth design

Study	Random assignment of respondents into groups	Concealment of randomization sequence	Blinding of participants/personnel	Blinding of outcome assessment	Selective reporting	Incomplete outcome data	Other bias sources	Overall risk of bias
Suarez et al. [9]	Yes	Yes	Unclear	Yes	Yes	Yes	Yes	Unclear
Spehar and Jakovac [17]	Yes	Yes	Yes	Yes	Yes	Yes	Yes	Low
Christensen and Ploeger [28]	Yes	Unclear	Yes	Yes	Yes	Yes	Yes	Unclear
Pelaez et al. [29]	Yes	No	Yes	Yes	Yes	Yes	Yes	High
Passia et al. [31]	Yes	Yes	Yes	Yes	Yes	Yes	Yes	Low
Sailer et al. [33]	Yes	Yes	Unclear	Yes	Yes	Yes	Yes	Unclear
Akin et al. [35]	Yes	Unclear	Yes	Yes	Yes	Yes	Yes	Unclear
Muhlemann et al. [36]	Yes	Yes	Yes	Yes	Yes	Yes	Yes	Low
Morsy et al. [37]	Yes	Unclear	Yes	Yes	Yes	Yes	Yes	Unclear

The Cochrane Collaboration Scale summarized an overall unclear risk of bias in most of the selected studies. For the RCTs, all nine studies reported using randomly assigned participants. Besides, these studies correctly reported outcome data and reported outcomes. These studies included all expected outcomes. These studies did not identify other biased sources. Most of the studies did not provide sufficient information about the concealment of the randomization sequence process to enable an assessment of a low or high risk of bias [28,29,35,37]. Others did not provide detail on the blinding of participants or personnel [9,33]. Only one study reported a high risk for the concealment of the randomization sequence process [29]. In summary, three studies had a low risk of bias, one had a high risk of bias, and five studies had an unclear risk of bias. Furthermore, based on the NOS scale, one study obtained a total score of 5 [2]. Two studies had a total score of 6 [30,34]. Only one study obtained a total score of 7 [32]. These scores indicate an acceptable quality of the studies being subjected to systematic review and meta-analysis.

Clinical Performance Encompassing Biological, Technical, and Esthetic Criteria

**Figure 2:**
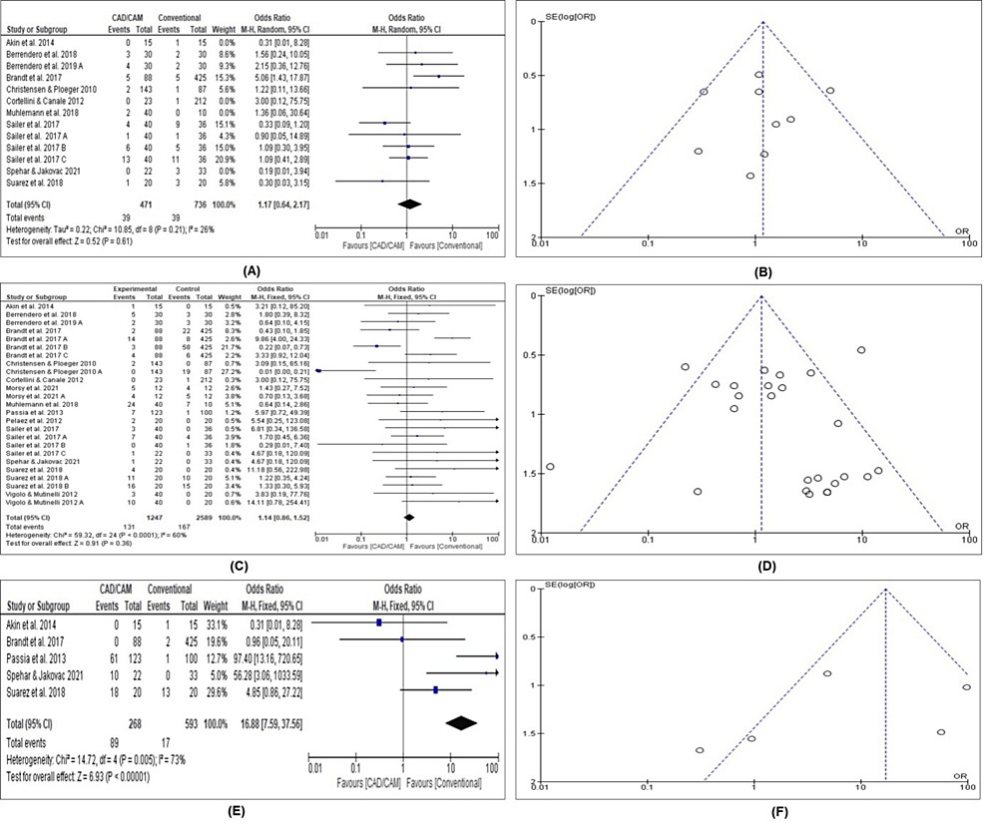
Clinical performance of all included studies (A) Forest plot of all biological outcomes [2,9,17,28,32-36] (B) Funnel plot of all biological outcomes (C) Forest plot of all technical outcomes [2,9,17,28-37] (D) Funnel plot of all technical outcomes (E) Forest plot of all esthetic outcomes [9,17,31,32,35] (F) Funnel plot of all esthetic outcomes.

The odds ratios for the clinical performance of CAD/CAM versus conventional for all the studies included in the meta-analysis are displayed in Figure 2.

In the forest plot for all biological outcomes (Figure 2A), the results of nine studies have been pooled, and a random-effects model has been fitted to the data. The estimated biological outcome over the mean observation period was 1.17 (95% CI: 0.64-2.17). Figure 2A indicates that more episodes of biological complications were seen in the CAD/CAM group than in the conventional group, with the difference between the two groups being statistically non-significant. The study by Brandt et al. could be considered to contribute the maximum weight to the meta-analysis for the biological outcomes [32]. According to the Chi-square test, there was no significant amount of heterogeneity in the true outcomes (Chi-square = 10.85, p = 0.21, I² = 26%), indicating consistent findings and a low suspicion for publication bias (Figure 2B).

Similarly, in the forest plot for all technical outcomes (Figure 2C), the results of 13 studies have been pooled in a fixed-effects model. The estimated technical outcome over the mean observation period was 1.14 (95% CI: 0.86-1.52). Figure 2C indicates that more episodes of technical complications were seen in the CAD/CAM group than in the conventional group; however, the difference between the two groups was statistically non-significant. Also, two studies were overly influential and contributed the most weight to the meta-analysis for the technical outcomes [28,32]. According to the chi-square test, there was a significant amount of homogeneity in the true outcomes (Chi-square = 59.32, p < 0.000, I² = 60%), indicating a low suspicion of publication bias (Figure 2D).

In the forest plot for all esthetic outcomes (Figure 2E), the results of five studies are pooled, and a random-effects model has been fitted to the data. The estimated esthetic outcome over the mean observation period was 16.88 (95% CI: 7.59-37.56). Figure 2E indicates that more episodes of the esthetic complications were seen in the CAD/CAM group than in the conventional group, with the differences between the two groups being statistically significant. Also, two studies contributed the most weight to the meta-analysis of the esthetic outcomes [9,35]. However, according to the chi-square test, there was a significant amount of heterogeneity in the true outcomes (Chi-square = 14.72, p = 0.005, I² = 73%), indicating a slight suspicion of publication bias (Figure 2F).

Clinical Performance Encompassing Biological, Technical, and Esthetic Aspects for SFCs and FPDs

**Figure 3:**
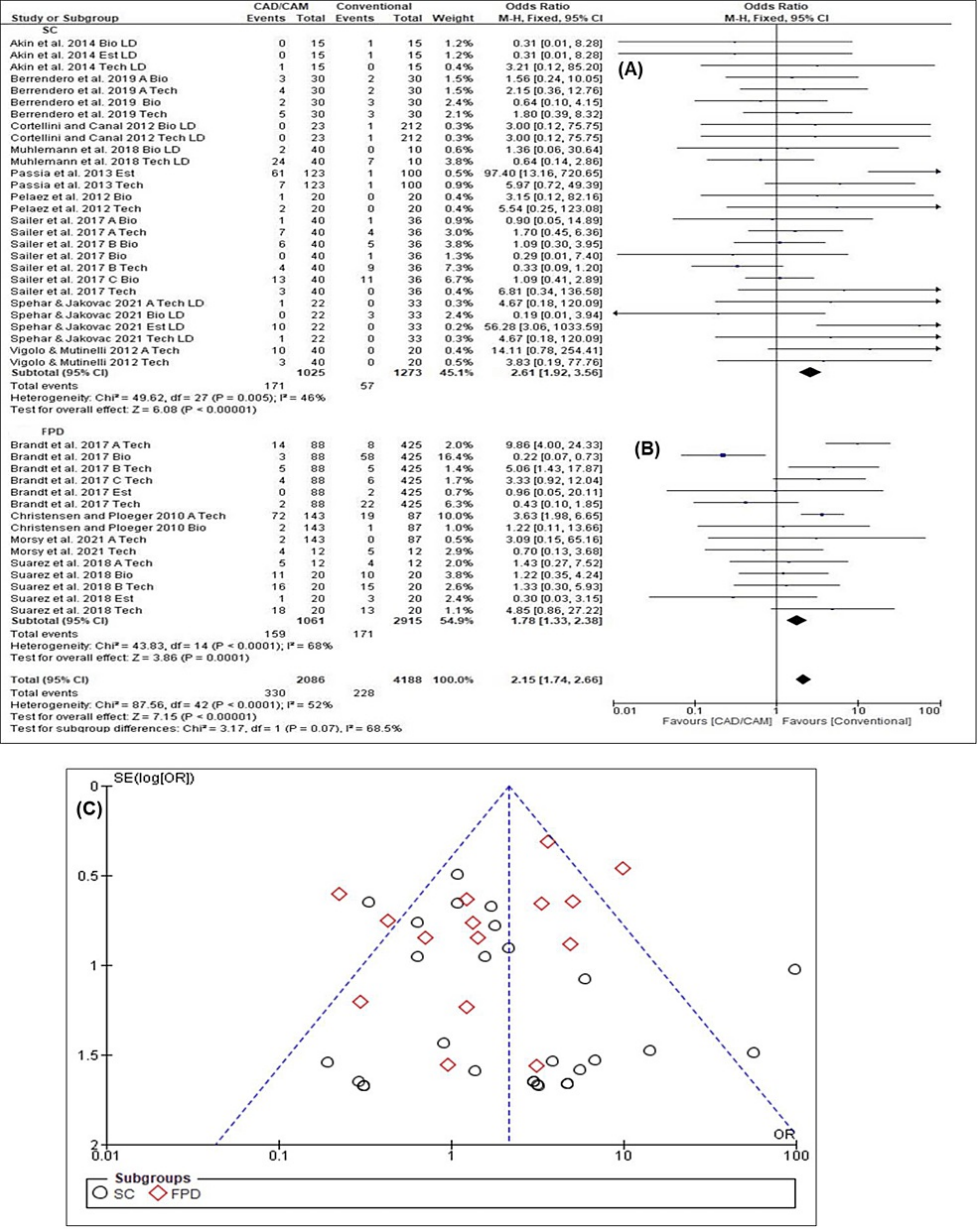
Clinical performance encompassing all biological, technical and esthetic aspects (A) forest plot for SFCs [2,17,29-31,33-36] (B) forest plot for FPDs [9,28,32,37] (C) funnel plot for SFCs and FPDs

The overall estimated clinical performance of all biological, technical, and esthetic aspects over the mean follow-up period was 2.15 (95% CI: 1.74-2.66). The clinical performance of SFCs was likely to be higher and was significantly different than that of FPDs (OR = 2.61 vs. 1.78, 95% CI: 1.92-3.56 vs. 1.33-2.38). The forest plot of SFCs (Chi-square = 49.62, I^2^ = 46.0%, p = 0.005) (Figure 3A) and FPDs (Chi-square = 43.83, I^2^ = 68.0%, p < 0.0001) (Figure 3B) showed significant homogenous findings, and the funnel plot (Figure 3C) exhibited a low possibility of publication bias. Overall, homogeneity was observed in the true outcomes between SCs and FPDs (Chi-square = 3.17, I^2 ^= 68.5%, p = 0.07).

Survival Ratios of Biological, Technical, and Esthetic Outcomes for SFCs and FPDs

**Figure 4:**
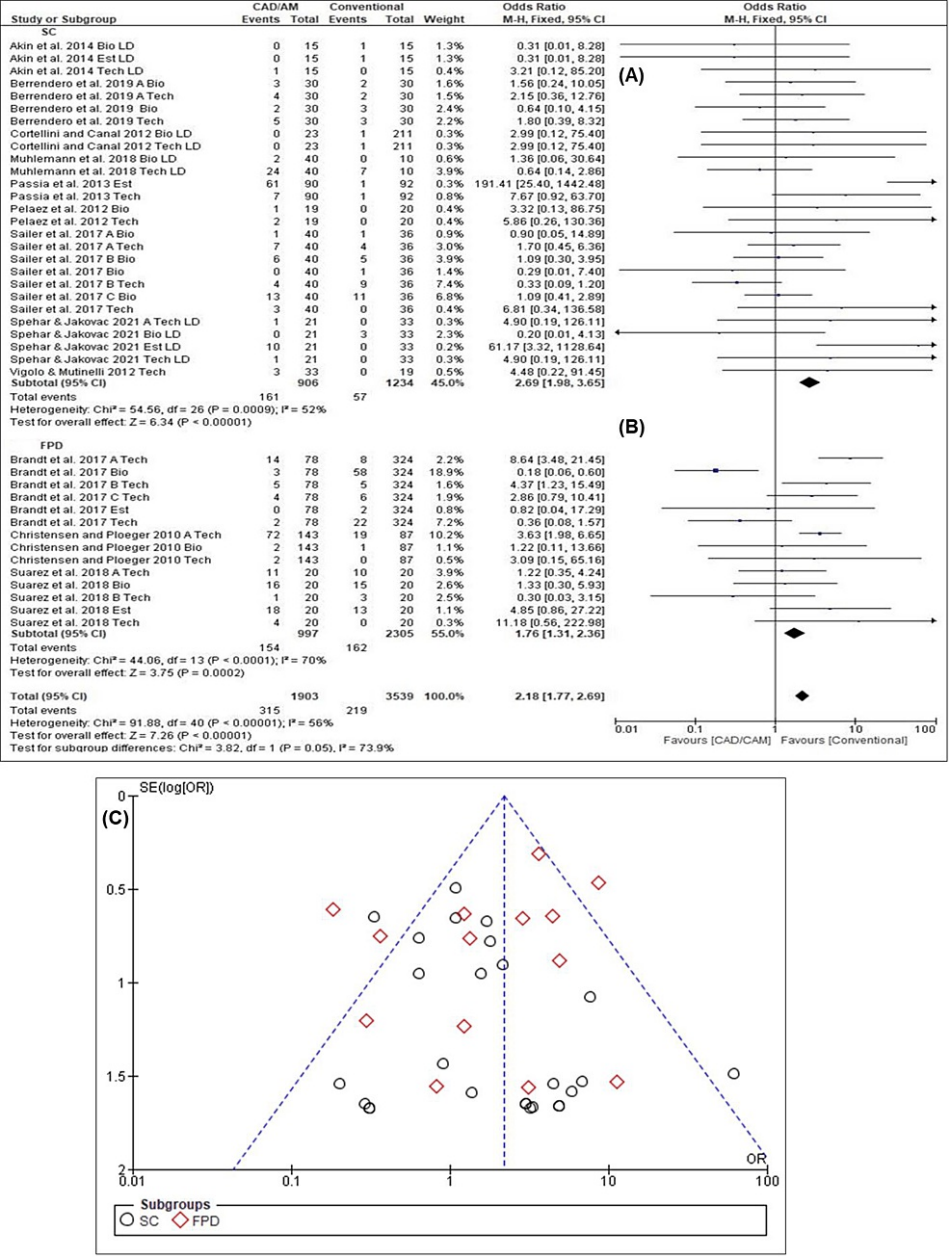
Survival ratios of all biological, technical and esthetic criteria (A) Forest plot for SFCs [2,17,29-31,33-36] (B) forest plot for FPDs [9,28,32,37] (C) funnel plot for SFCs and FPDs

The overall estimated survival ratio over the mean observation period was 2.18 (95% CI: 1.77-2.69). The survival ratios of SFCs (OR = 2.69, 95% CI: 1.98-3.65) were significantly higher than those of FPDs (OR = 1.76, 95% CI: 1.31-2.36). The forest plot of SFCs (Chi-square = 54.56, I^2^ = 52.0%, p = 0.0009) (Figure 4A) and FPDs (Chi-square = 44.06, I^2^ =70.0%, p < 0.0001) (Figure 4B) revealed homogeneous results, while the funnel plot (Figure 4C) exhibited a possibility of publication bias for FPDs and a low possibility of publication bias for SFCs. Overall, homogeneity was observed in the survival ratios between the SFCs and FPDs (Chi-square = 91.88, I^2^ = 56.0%, p < 0.00001).

S*uccess Ratios of Biological, Technical, and Esthetic Outcomes for SFCs and FPDs*

**Figure 5:**
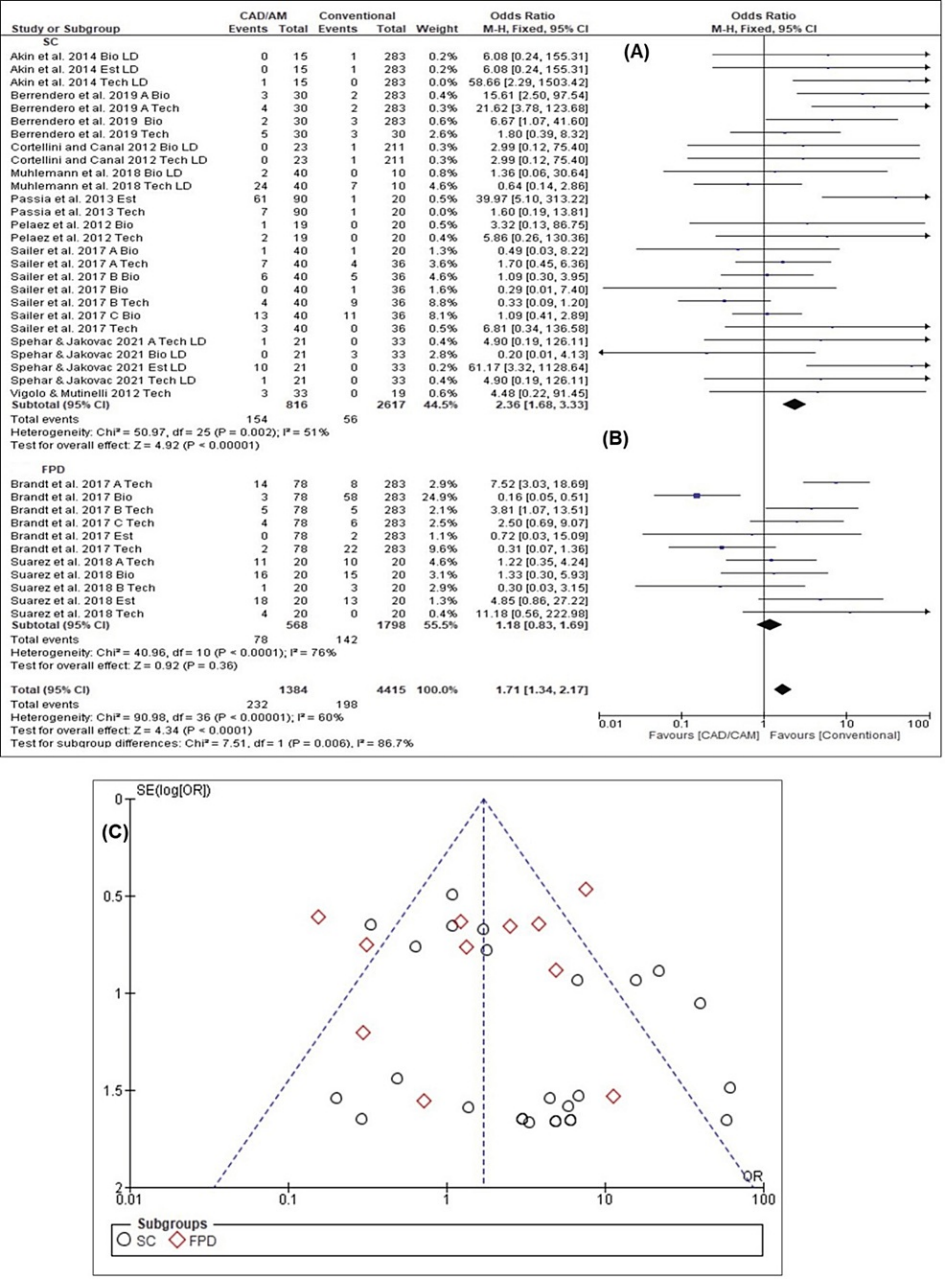
Success ratios of all biological, technical and esthetic criteria (A) Forest plot for SFCs [2,17,29-31,33-36] (B) forest plot for FPDs [9,28,32,37] (C) funnel plot for SFCs and FPDs

The overall estimated success ratio over the mean observation period was 1.71 (95% CI: 1.34-2.17). The success ratios of SFCs (OR = 2.36, 95% CI: 1.68-3.33) were higher than those of FPDs (OR = 1.18, 95% CI: 0.83-1.69). Results of the forest plot for SFCs (Chi-square = 50.97, I^2^ = 51.0%, p = 0.002) (Figure 5A) revealed significant homogeneous results, while the forest plot for FPDs (Chi-square = 44.96, I^2^ =76.0%, p < 0.0001) (Figure 5B) revealed significant heterogeneity. Moreover, the funnel plot (Figure 5C) exhibited a low possibility of publication bias for SFCs and a possibility of publication bias for FPDs, since there was a slight hint of too-high success ratios. Overall, homogeneity was observed in the success ratios between the SFCs and FPDs (Chi-square = 90.98, I^2^ = 60.0%, p < 0.00001).

Clinical Performance Encompassing Biological, Technical, and Esthetic Aspects for LD- and ZC-Based Restorations

**Figure 6:**
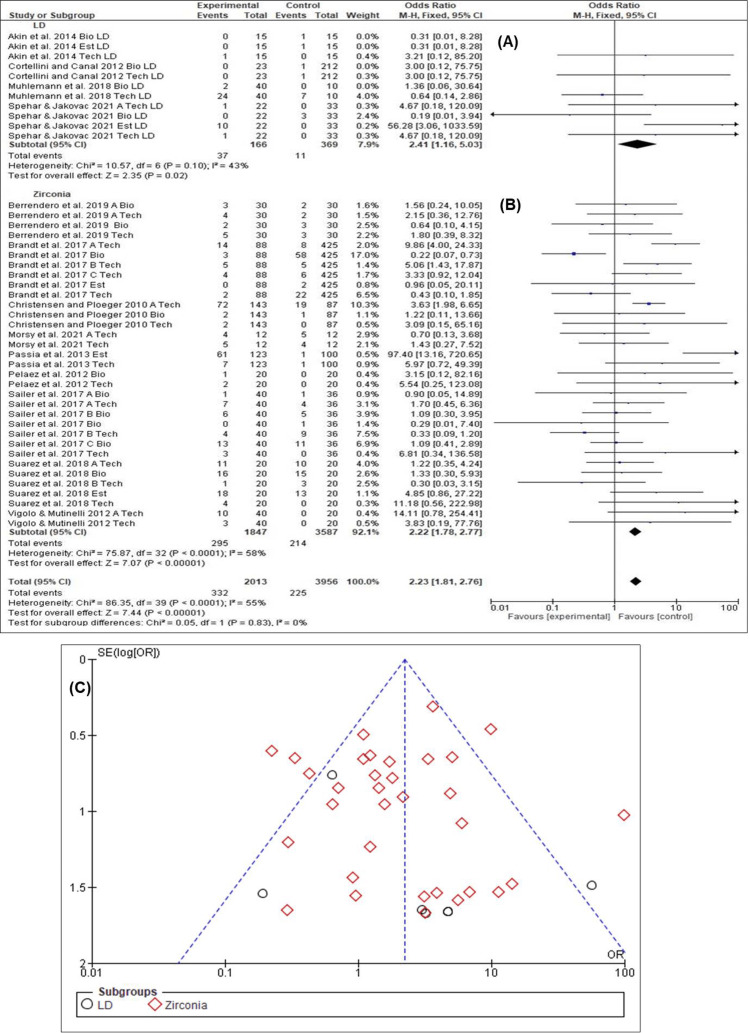
Clinical performance of lithium disilicate (LD) and zirconia (ZC)-based restorations (A) Forest plot for LD [17,34-36] (B) Forest plot for ZC [2,9,28-33,37] (C) funnel plot for LD and ZC

The odds ratios for the clinical performance between LD and ZC restorations for all the studies included in the meta-analysis are displayed in Figure 6. Consolidating the data with a random-effects model to explain the assessment of the type of restoration materials, the overall estimated clinical performance of all biological, technical, and esthetic criteria for LD and ZC restorations over the mean observation period was 2.23 (95% CI: 1.81-2.76). A significant difference (p<0.00001) was recorded between the fabricated materials used. LD-based CAD/CAM restorations showed better clinical performance (OR = 2.41, CI: 1.16-5.03) compared to ZC-based restorations (OR = 2.22, CI: 1.78-2.77). Results of the forest plot for LD-based restorations (Chi-square = 10.57, I^2^ = 43.0%, p = 0.10) (Figure 6A) and for ZC-based restorations (Chi-square = 75.87, I^2^ = 58.0%, p<0.0001) (Figure 6B) revealed homogeneous findings. Moreover, the funnel plot (Figure 6C) exhibited a low possibility of publication bias for the two groups.

Discussion

The present study investigated the clinical performance, survival, and success ratios of all biological, technical, and esthetic aspects of CAD/CAM and conventionally manufactured SFCs and FPDs, according to the materials used (LD and ZC) in the existing studies. Since these materials and techniques have been developed recently, their clinical indications are still being evaluated. Most of the existing studies reported on CAD/CAM and conventional techniques’ survival and clinical complication rates [7,38,39]. However, only a few studies compared CAD/CAM and conventional manufacturing of restorations regarding the survival and success of all biological, technical, and esthetic complications [23,40]. To the best of the authors’ knowledge, the present study is among the first to compare LD- and ZC-based CAD/CAM and conventionally manufactured SCs and FPDs in terms of clinical performance, survival, and success ratios of all biological, technical, and esthetic aspects over a minimum of one year of follow-up time. The meta-analysis of the included studies reported the outcomes as the odds ratio, which was calculated as the ratio of the odds of clinical performance, survival, and success of all biological, technical, and esthetic aspects in the CAD/CAM group to those in the conventional group, thus providing consolidated values for the outcomes. A funnel plot was used to detect any risk of publication bias among the studies; none were observed for biological and technical outcomes and survival ratios of all biological, technical, and esthetic aspects for SFCs and FPDs, while a publication bias was likely observed for clinical performance and success ratios of all esthetic outcomes for FPDs (Figures 2F, 5C).

The 13 studies selected for the meta-analysis indicated that conventional restorations resulted in better clinical performance compared to CAD/CAM restorations for biological, technical, and esthetic complications. This finding is consistent with previous studies that suggested that the clinical performance and longevity of CAD/CAM restorations were lower compared to conventionally manufactured restorations [38,41,42]. This could be attributed to the fact that all the studies included in the meta-analysis used veneering ceramic frameworks; only one used a monolithic ceramic framework. In a recent study by Elshiyab et al., monolithic frameworks were reported to have no technical complications in comparison to veneering frameworks [43]. Although the information on monolithic ceramic restorations is still not adequate, these are considered effective options for mitigating or eliminating the increased incidence of technical complications. Moreover, the lack of adequate experience with ceramics, improper framework support, the thickness of the veneer layer, cooling rate inaccuracies in firing schedules, and surface damage from digital techniques might be the usual causes of clinical complications in CAD/CAM-based restorations [15]. Besides, the increased incidences of clinical complications among the CAD/CAM systems might be due to the type of ceramics and their properties and the internal integrity between the tooth and restoration [14]. Comparing the clinical aspects, the predominant technical complications for CAD/CAM restorations were chipping, framework fracture, marginal gap, anatomical shape, loss of retention and absence of interproximal contact point, insufficient restoration margin, and internal gap. Among these complications, chipping, framework fracture, marginal gap, and loss of retention were more often encountered for the CAD/CAM SFCs and conventional FPDs. This is in corroboration with previous studies where chipping of veneering material in FDPs was noted as the most frequent technical complication [38,44]. Also, conventionally manufactured restorations may have reduced incidences of chipping issues due to the framework-veneer interface [45]. Besides, another study by Sailer et al. reported framework fracture, ceramic fracture, and retention loss as the recurrent reasons for technical complications [7]. Chipping in FDPs has been often reported as a problem since the initiation of this restoration [46]. Moreover, the most frequent biological complications for CAD/CAM full crowns were periodontal inflammation, secondary caries, hypersensitivity, occlusal wear, and approximal contact defect, and for conventional FPDs, periodontal and pulpal inflammations, dental caries, apical osteitis, and root fracture. This finding is congruent with the study by Sailer et al., which reported secondary caries as the most frequent biological complication encountered with SFCs [7]. However, Pjetursson et al. reported the loss of tooth vitality as the most recurrent biological complication observed with SFCs [6]. This variation in results could be due to the type of material used, the technique used, or the tooth position [47]. Moreover, marginal discoloration was reported as the most frequent esthetic complication in this study. This could be attributed to the deterioration of the restorative material over time [48]. Furthermore, ‘fracture’ of the CAD/CAM-based restorations was seen to be the most frequent cause of failure in this study, which is consistent with the previous studies where the fracture was identified as the most frequent reason for the failure of CAD/CAM restorations [49,50]. This could be attributed to the fact that most ceramic materials are susceptible to fracture. So far, conventional metal ceramics have been considered the ideal fabrication material for FDPs and SFCs [51].

Moreover, this study reported that SFCs showed overall better clinical performance than the FPDs based on biological, technical, and esthetic aspects. Similar findings were found for survival rates based on the overall clinical performance of full crowns (99%) and the 5-year survival rates of SFCs for glass-ceramic reinforced with leucite or LD (96.6%) and zirconia (96%) [7]. This study assessed the survival and successes of all biological, technical, and esthetic aspects and observed that the survival and success ratios of SFCs were higher compared to FPDs. The meta-analysis found a significant difference between the groups being compared. However, in some studies, the survival and success rates were comparable over the observation period for both CAD/CAM and conventional restorations. This could be due to the improved bond strength with the conventional technique and the consistency of the CAD/CAM ceramic restoration [6]. Current trends for the selection of materials in the fabrication of dental restorations highlight the inclination towards non-metallic and esthetic materials among patients and clinicians. However, concerning SFCs, existing studies still support PFM crowns as the ideal option due to their persistent survival rates of more than 95% [7,52]. Moreover, studies concerning longevity demonstrated that CAD/CAM ceramic full crowns have lower survival rates compared to conventionally manufactured ones [38]. The findings of previous studies related to the survival rates of LD (96.6-95%) and ZC (91.2%) SFCs are consistent with the present study, which demonstrated better clinical performance for LD-based CAD/CAM restorations compared to ZC-based restorations [7]. Lately, ZC has been increasingly used as an alternative to conventional ceramic materials because of its lower price and higher esthetic value [46]. Further advances in ZC and the veneering procedure have helped alleviate the originally high frequencies of chipping, but the problem remains persistent. However, only one study comparing CAD/CAM-based monolithic ZC and conventional FPDs was available for the meta-analysis with an observation period of one year [37]. As such, repeating the meta-analysis when more information is available is recommended to assess the above supposition.

The reviewed articles included in the present study used the USPHS standard to evaluate the clinical performance of the SFCs and FPDs over the follow-up periods. These criteria were also used in previous reviews and meta-analyses [24,48]. Although such criteria are used to normalize the assessment of dental restorations, not all the included studies assessed the clinical performance based on these criteria. Moreover, most of the articles analyzed the clinical performance related to two different materials mentioned for SFCs or FPDs within the same study or compared the same material, differentiating the manufacturing techniques.

Nevertheless, the present study had some limitations, and thus the results need to be considered keeping this in mind. First and foremost, the numbers of CAD/CAM SFCs and FPDs fabricated from LD or ZC selected for this meta-analysis were different. More studies comparing the CAD/CAM group and conventional group for ZC-based restorations were available for this meta-analysis. Three studies on monolithic LD SFCs were included [17,34,36]. Only one study on monolithic ZC-based FPD could be included in the meta-analysis [37]. Thus, the interpretation of the findings is mostly confined to veneered ZC and LD. Future studies should emphasize novel CAD/CAM-based monolithic ceramic restorations to compare their outcomes with conventional restorations. Furthermore, the findings of this study reported the lack of a consistent number of restoration material type groups. More homogeneous studies with more comparable restorative materials and CAD/CAM techniques, a control group in a split-mouth RCT, and prospective cohort studies should be conducted.

## Conclusions

The findings of the present study suggest that the biological, technical, and esthetic behaviors showed similar clinical outcomes, with more biological, technical, and esthetic complications being observed in the CAD/CAM group than in the conventional group. However, a significant difference was found in the aesthetic aspects only. Studies that assess the software limitations in CAD/CAM should be considered to explain the reasons behind the clinical complications with CAD/CAM restorations. The clinical performance of SFCs was higher than that of FPDs, and these comparisons were significant for both groups. Both the survival and success ratios of all biological, technical, and esthetic aspects for SFCs were higher than for FPDs, and these comparisons were significant for both groups. The clinical performance of LD in terms of all biological, technical, and esthetic aspects was significantly better than that of ZC. Therefore, LD could be a good alternative, but its intermediate or persistent biological, technical, and esthetic complications need to be evaluated and considered while addressing dental restorations. Altogether, presently, zirconia and CAD/CAM techniques must evolve further to outclass the conventional techniques used in the fabrication of SFCs and FPDs. However, more RCTs and prospective cohort studies are needed to strengthen the findings of the study.
